# Supplementary material for: Oleogel-Based Nanoemulsions for Beverages: Effect of Self-Assembled Fibrillar Networks on Stability and Release Properties of Emulsions
Source: Foods. 2024 Feb 23;13(5):680. doi: 10.3390/foods13050680 (PMC10930539; doi:10.3390/foods13050680)
Supplement: Supplementary file 1 [file foods-13-00680-s001.zip › foods-2864129-supplementary.pdf]

Optimization of process parameters

Optimizing sonication amplitude and time

An increase in amplitude from 30 to 50 % produced an exponential decrease in size (D_{avg}) and PDI of the particles in both the NEs (Fig. S1a and b). Upon further increase in amplitude to 70 %, the decline had a minimum effect i.e., relative saturation was observed. This kind of saturation was also observed during the development of paraffin wax NEs (1). The lowered effect can be attributed to the loss of emulsifier activity due to the increase in temperature from the high collision frequency between the dispersed droplets (2). This is evident with the phase separation (vial inserts in Fig. S1a and b). At lower amplitudes (30 % and 50 %), optimal acoustic cavitation is created which in turn reduced the dispersion size. Relatively, more stable emulsions were formed when the sonication amplitude was at 50 %.

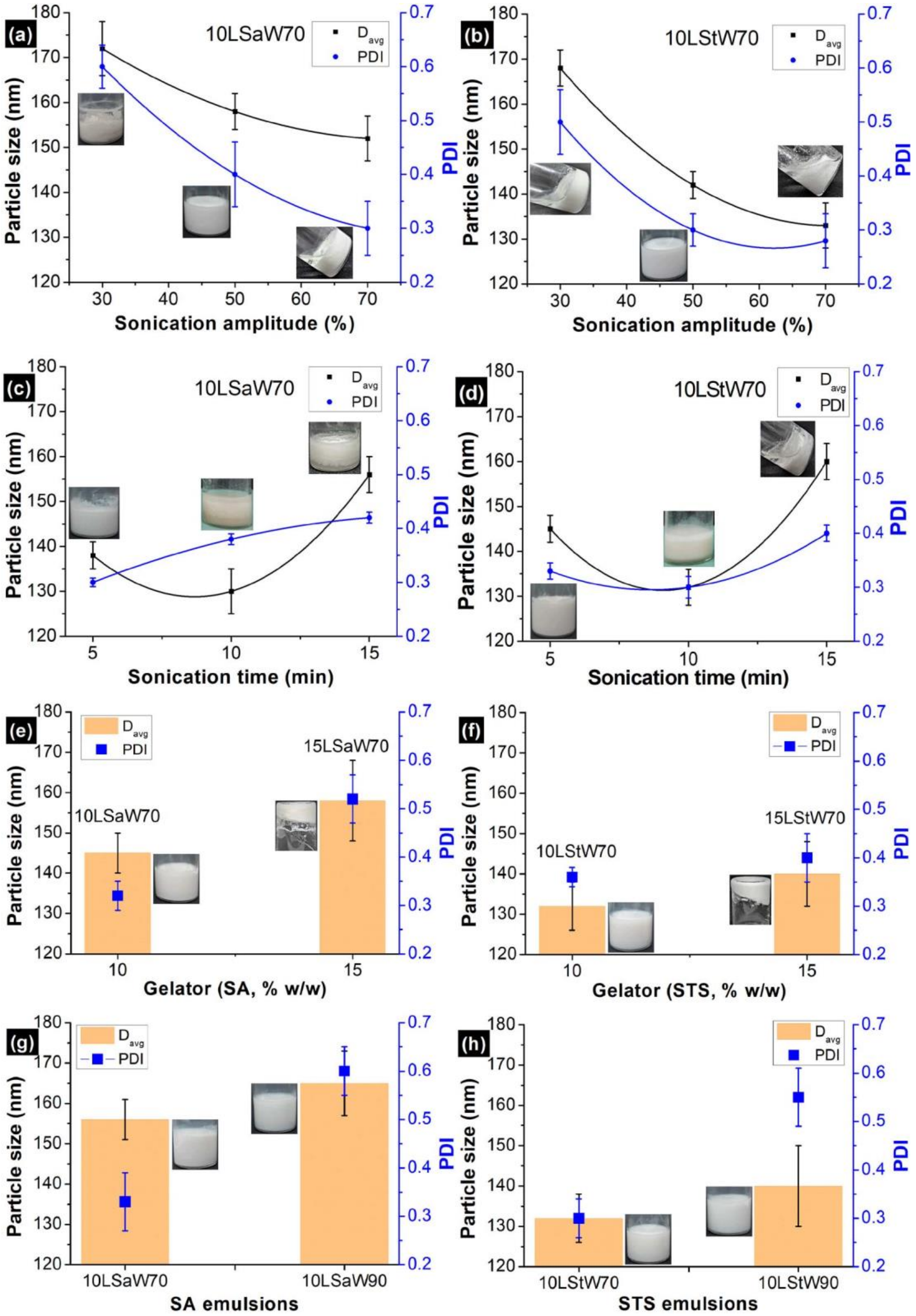

Fig. S1. Optimizing the (a, b) sonication amplitude; (c, d) sonication time, (e, f) gelator concentration and (g, h) aqueous fraction to form stable NEs.

At the optimal amplitude (50 %), three sonication times (5, 10 and 15 min) were tested (Fig. S1c and d). The D_{avg} decreased with the increase in time to 10 min, beyond which an exponential increase in size was observed in both the emulsions. Creaming was noticed in all the samples when 10 or 15 min of sonication was done (insert vials in Fig. S1c and d). Homogenization for excess period of time could result in creaming (3). On the other hand, PDI did not follow the similar pattern, although an overall increase was observed in both the emulsions. In general, it is expected to have narrow size distribution of the dispersed phase with increasing sonication time. Longer sonication times allow the droplets to pass through the disruption zone to facilitate higher fragmentation (2,4). However, this behavior holds true up to a certain limit of sonication time. As observed with amplitude, prolonged sonication times also damage the efficiency of emulsifiers (5). Here, both the gelator networks are thermosensitive; henceforth unstable emulsions were formed at higher sonication amplitude and time. Along with the higher amplitude, signs of were seen when 50 % amplitude was applied for either 10 or 15 min. This study proves that sonication must be done at 50 % amplitude for 5 min to achieve stable NEs.

Optimizing the gelator concentration

Although 5LSt is unstable, its emulsification ability was tested along with the stable oleogels by incorporating 70 % w/w water. After sonication under the optimized conditions, phase separation was noticed in 5LSa- and 5LSt-based emulsions (Fig. S2). Though 5LSa is a stable oleogel, its emulsification ability happened to be poor. Lack of sufficient gelator networks and poor emulsification seem responsible for the instability of emulsions. Larger standard deviation in size and PDI of the emulsions with higher gelator concentration (15 % w/w) indicate non-uniform mixing (Fig. S1e and f). Observing oil syneresis upon vial inversion further confirms the poor homogenization in both 15LSaW70 and 15LStW70 (vial inserts in Fig. S1e and f). Higher gelator fraction enhanced the viscoelasticity of these emulsions; thereby droplets disruption was hindered. Another possible reason for the increment in particle size with 15LSaW70 and 15LStW70 would be that the rate of collision frequency might be higher with higher dispersion volume and thus coalescence rate was increased (5). In this regard, 10LSa and 10LSt oleogels were chosen to study the effect of water concentration during emulsification.

Optimizing the aqueous fraction

When water fraction was varied under the optimized sonication conditions (amplitude: 50 %; time: 5 min), stable liquid emulsions were formed at 70 and 90 % (w/w) in both the types of oleogels (Fig. 1b). When water fraction was low (30 and 50 % w/w), non-uniform, semi-solid emulsions were formed, except 10LStW50 (Fig. 1b). Colloidal stability of the emulsions is given in Table 2. Gel-like aggregates or clumps in emulsion gels (emulgels) can be attributed to the formation of aggregated particles (6). For better comparison, NEs with higher water fraction (70 and 90 % w/w) were chosen. The particle size and PDI were found to be high in emulsions with higher (90 % w/w) aqueous fraction ($P > 0.05$) (Fig. S1g and h). The lower gelator fraction in 10LSaW90 and 10LStW90 might have allowed droplets coalescence and resulted in larger size and PDI. Smaller standard deviation in samples containing 70 % (w/w) water indicate the occurrence of better emulsification. This study indicates that 10LSaW70 and 10LStW70 NEs are more stable by having better size and PDI compared to the NEs with 90 % (w/w) aqueous fraction.

The stability of NEs was further determined by measuring ζ potential (Fig. S3). Lower ζ potential in 10LSaW90 and 10LStW90 confirm the presence of less amount of SAFiNs around the dispersed oil fraction. The increase in gelator concentration has increased the ζ potential, suggesting the prevalence of more gelator networks at the interface. This also indicate that gelator networks are stabilizing the gelled-oil dispersion by creating stearic hindrance (7). The observed negative charge can be attributed to lecithin. The sorbitol moiety of STS also contributed to the negative charge, henceforth LSt NEs showed better ζ potential.

Based on this study, the optimized sonication and formulation condition for stable 10LSa and 10LSt NEs is 50 % amplitude for 5 min of time in the emulsion containing 70 % (w/w) water fraction.

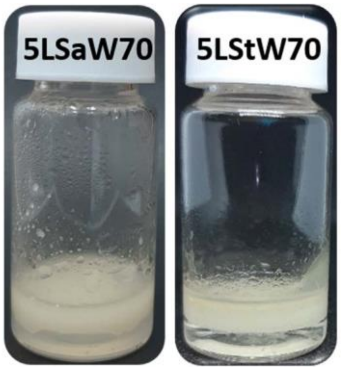

Fig. S2. 5LSa and 5LSt oleogel-based unstable emulsions

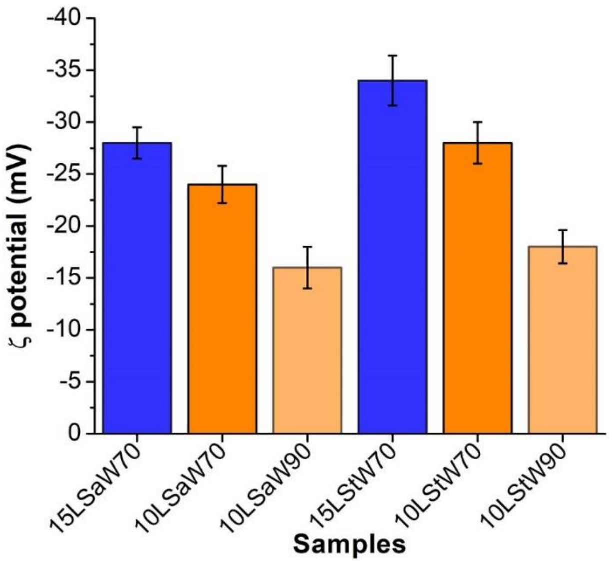

Fig. S3. ζ potential of 10LSa and 10LSt-based stable emulsions

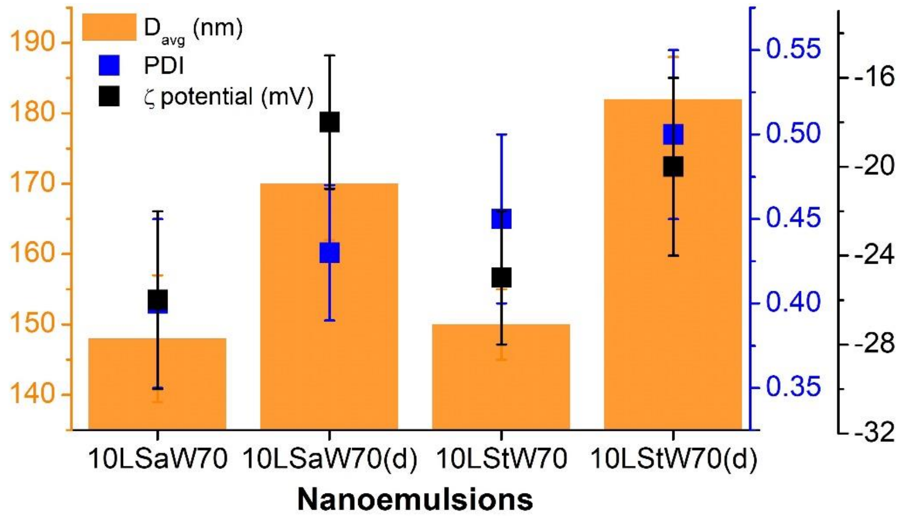

Fig S4. D_{avg}, PDI and ζ-potential of concentrated and diluted NEs after six months of storage

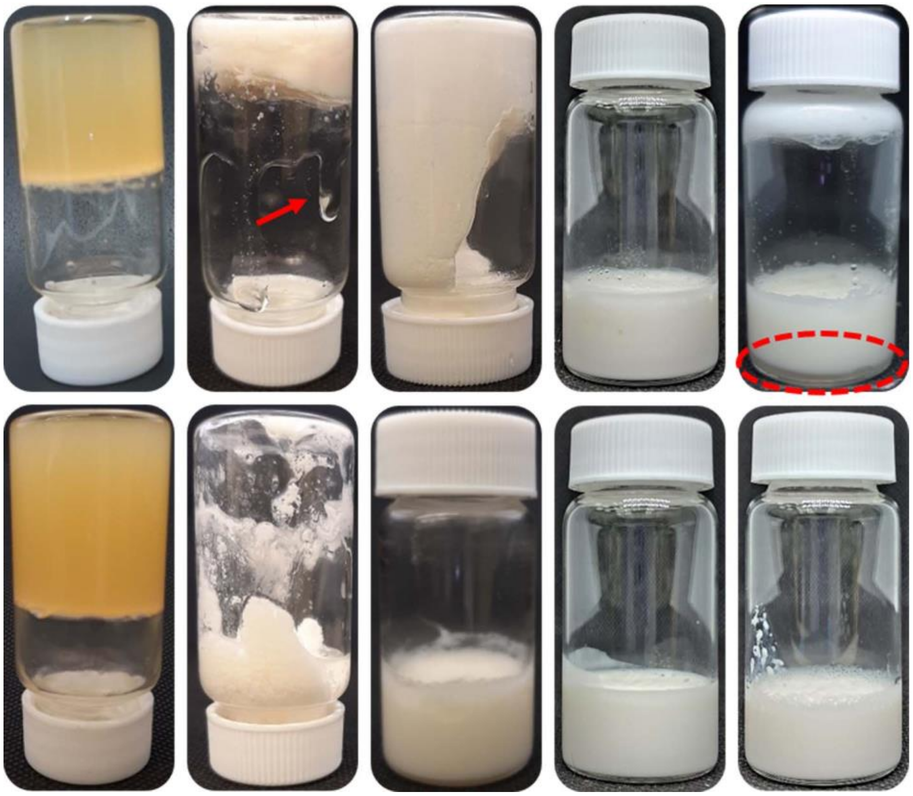

Fig. S5. Vials of 10LSa (top row) and 10LSt (bottom row) oleogels and the corresponding nanoemulsions after six months of preparation. Top row from left to right: 10LSa, 10LSaW30, 10LSaW50, 10LSaW70, and diluted 10LSaW70. Bottom row from left to right: 10LSt, 10LStW30, 10LStW50, 10LStW70, and diluted 10LStW70. The arrow indicates oil syneresis and the dotted circle shows the phase separation.

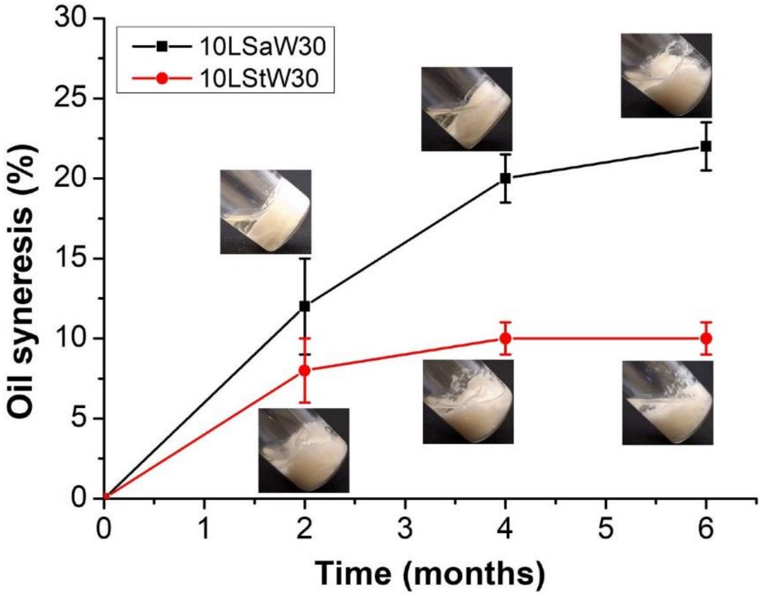

Fig. S6. Quantification of oil syneresis from emulgels

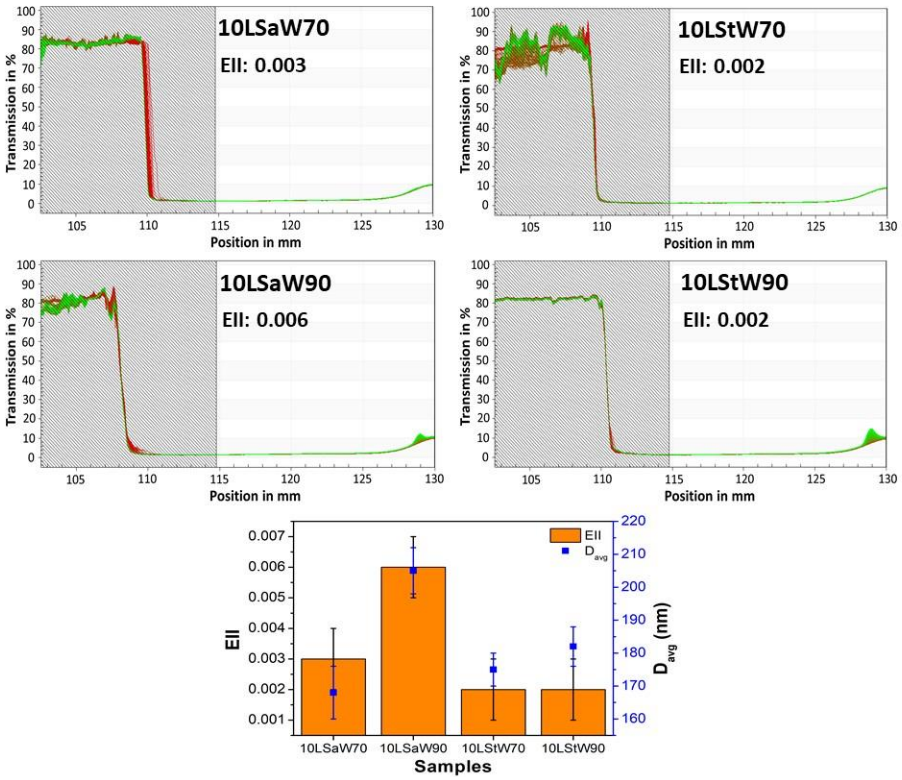

Fig. S7. Lumisizer profiles of concentrated NEs and their instability index (EII) along with the size of the dispersed phase

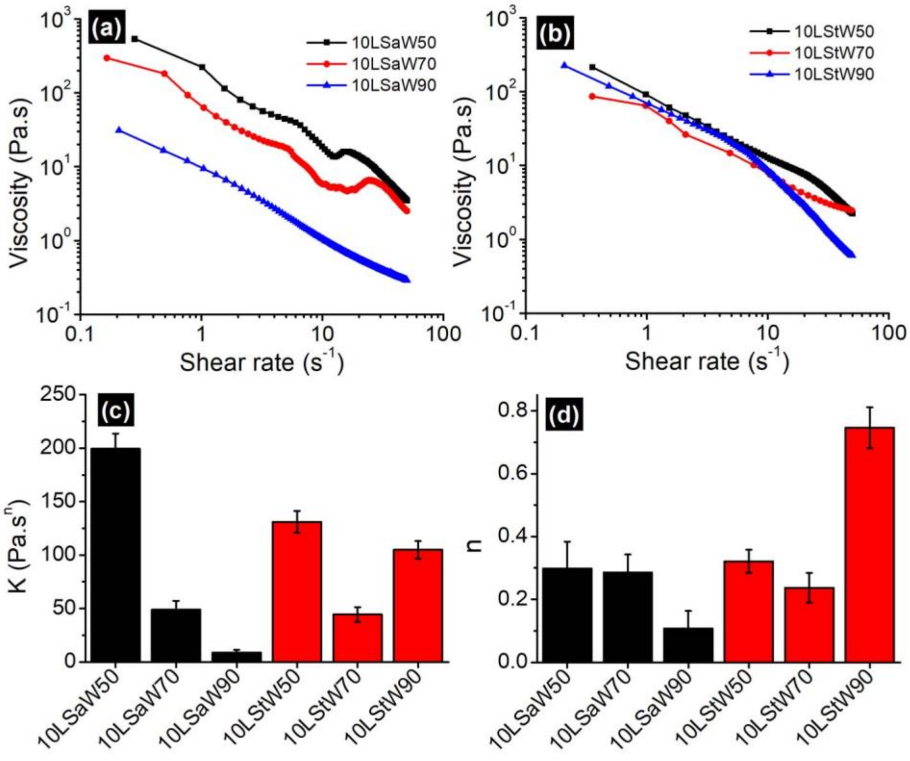

Fig. S8. Apparent viscosity profiles of (a) 10LSa- and (b) 10LSt-based emulsions. The flow parameters (K, n) after fitting the data in modified power law are shown in (c) and (d).

References

1. Jadhav AJ, Holkar CR, Karekar SE, Pinjari DV, Pandit AB. Ultrasound assisted manufacturing of paraffin wax nanoemulsions: Process optimization. Ultrasonics Sonochemistry. 2015 Mar 1;23:201–7.

2. Gharibzahedi SMT, Jafari SM. Chapter 9 - Fabrication of Nanoemulsions by Ultrasonication. In: Jafari SM, McClements DJ, editors. Nanoemulsions [Internet]. Academic Press; 2018 [cited 2022 Jul 15]. p. 233–85. Available from: https://www.sciencedirect.com/science/article/pii/B9780128118382000096

3. Sui X, Bi S, Qi B, Wang Z, Zhang M, Li Y, et al. Impact of ultrasonic treatment on an emulsion system stabilized with soybean protein isolate and lecithin: Its emulsifying property and emulsion stability. Food Hydrocolloids. 2017 Feb 1;63:727–34.

4. Shahavi MH, Hosseini M, Jahanshahi M, Meyer RL, Darzi GN. Evaluation of critical parameters for preparation of stable clove oil nanoemulsion. Arabian Journal of Chemistry. 2019 Dec 1;12(8):3225–30.

5. Jafari SM, Assadpoor E, He Y, Bhandari B. Re-coalescence of emulsion droplets during high-energy emulsification. Food Hydrocolloids. 2008 Oct 1;22(7):1191–202.

6. Ghiasi F, Eskandari MH, Golmakani MT, Hosseini SMH. Development of highly stable colloidal dispersions of gelled-oil nanoparticles loaded with cuminaldehyde. Journal of Colloid and Interface Science. 2019 Apr 1;541:65–74.

7. Magri A, Petriccione M, Cerqueira MA, Gutiérrez TJ. Self-assembled lipids for food applications: A review. Advances in Colloid and Interface Science. 2020 Nov 1;285:102279.
